# Social distancing intentions to reduce the spread of COVID-19: The extended theory of planned behavior

**DOI:** 10.1186/s12889-021-11884-5

**Published:** 2021-10-11

**Authors:** Wignyo Adiyoso, Wilopo

**Affiliations:** 1 National Development Planning Agency/BAPPENAS, Jl. Proklamasi No. 70, Jakarta Pusat, Indonesia; 2 Research Centre for Conflict and Policy (RCCP), Faculty of Administrative Science, Brawijaya University, Jl. MT. Haryono 163, Malang, East Java Indonesia

**Keywords:** COVID-19, Social distancing, Theory of planned behavior, Risk perception, Health risk, Media use

## Abstract

**Background:**

Due to the absence of a vaccine of SARS-CoV-2 virus pandemic, the most effective way to reduce transmission of the virus is by applying social distancing practices. Exploring factors that determine whether people adopt social distancing measures is, therefore, critical to reducing the spread of the virus. This study aimed to investigate people’s intentions to socially distance based on the extended Theory of Planned Behavior.

**Methods:**

A questionnaire was distributed to the sample population and collected through social media online and WhatsApp groups from March 26, 2020 to March 29, 2020. There were 823 analyzed out of 1870 responses. The extended TPB variables and risk perception were measured using a 7-point scale (scored from 1 to 7). Data were analyzed using the partial least squares (PLS) structural equation modeling method.

**Results:**

Study found that the factors influencing the intention to perform social distancing were subjective norms and perceived behavior control. Risk perception affected attitudes, subjective norms, and perceived behavioral control. Media exposure was found to determine attitudes, subjective norms, and risk perceptions. The relationship between attitudes and intentions, and media use and perceived behavior control were not significant. The better and effective risk communication that can change the level of risk perception, raise family and religious leader as well as increase to control behavior are the keys to people’s perform social distancing. Results from a multigroup analysis revealed that younger individuals showed a stronger relationship between the influence of risk perception and PBC and media use on risk perception. The predictive strength of subjective norms from risk perception and risk perception from media use was more strongly associated with rural populations.

**Conclusions:**

The results of study provide an initial understanding of the level of the public’s risk perception to reduce the spread of SARS-CoV-2. Findings also revealed the role of media use in determining risk perception, attitudes and subjective norms and, in turn, change people’s intention to socially distance. This study may add to the literature of behavioral changes in pandemic and provide a framework for both policymakers and practitioners to formulate effective interventions in the future.

**Supplementary Information:**

The online version contains supplementary material available at 10.1186/s12889-021-11884-5.

## Background

Since the first case of COVID-19, the disease caused by the novel coronavirus SARS-CoV-2 that was first reported in December 2019 in Wuhan, Hubei Province, China, the virus has achieved a global spread. While China has reported a significant reduction of new cases, the outbreak continues to escalate in other parts of the world [1]. The virus is especially difficult to control as many infected individuals experience only mild to moderate respiratory symptoms and recover with no medical treatment [2]. Despite efforts to develop an effective treatment for COVID-19 and a vaccine against the virus, currently there is nothing yet available that has been shown to be effective. Public officials in the United States have suggested that a vaccine against Sars-CoV-2 will likely be available in 12 to 18 months [3] [4];.

While the public awaits a vaccine, public health measures need to be taken to stop the spread of SARS-CoV-2. To both prevent and slow transmission of the virus, the WHO has recommended protective behaviors such as regularly washing hands with soap and water or alcohol-based hand sanitizers, keeping a distance of at least 1 meter from others, avoiding crowded places, covering the mouth and nose when coughing, staying at home or self-isolating at home, and practicing physical activities [5, 6]. Governments have disseminated information and encouraged people to stay at home through television, newspapers, internet-based media, and social media. However, hoaxes, rumors, and false information are also spreading through social media, which has influenced the public’s perception of COVID-19.

Although the majority of people strongly support social distancing practices, the number of people who refuse to socially distance remains high. For example, a Politico/Morning Consult Poll involving 1991 people conducted from April 21–26, 2020 in the United States revealed that 73% of respondents socially distanced, with 15% refusing to engage in the practice [7]. In India, Bangladesh, Pakistan, and Indonesia people continue to go to shopping malls, houses of worship, tourism destinations, as well as continue to use public transport [8, 9], which are activities that make social distancing difficult. As governments decide not to impose lockdown policies, effective control over the spread of the virus and minimizing the effects of the pandemic will be dependent on public behavior. Exploring factors that determine whether people adopt social distancing measures is, therefore, critical to reducing the spread of the virus. As Indonesia is one the most densely populated and diverse countries in terms of culture, race, language, and religion, the population is especially vulnerable to COVID-19.

There has been a growing body of theory, research, and application regarding human behavior. Behavior and behavior changes have become a particular topic and attract the attention of scholars because it is important to understand and predict human behavior [10]. Many studies focused on investigating factors causing personal behavior such as health behavior [11], disaster preparedness [12], election, and environmental protection [13, 14]. Public health authority, for example, needs to know the reasons for people to quit or not to quit smoking in order to make intervention policies in reducing the negative impact of smoking. In regard to the importance of understanding and predicting behavior change, significant midrange theories exist and can be divided into some clusters such as behavior or individual model, communities or cultures model, and social cognition model [15].

One theory that comes from the individual model is Rational Choice Theory (RCT). The RCT posits that every choice is based on assessing the costs, risks, and advantages of making that option [16]. Similar to the RCT is the Fogg Behavior Model (FBM), which claims that individual behavior is influenced by motivation, ability, and trigger components that occur together at the same time [17]. Instead of depending on individual motive, the social learning theory introduced by Albert Bandura [18] highlighted that people’s behavior is a process of observing and modeling the behavior, attitude, and emotional reactions. It concerns learning that takes place in a social setting.

Behavioral theories focusing on the social cognition model have been widely used and become the foreground of research into predicting and explaining health behavior [19], social marketing [15], and lifestyle [20]. According to Goldberg et al., [15], they include the Theory of Reasoned Action (TRA), Theory of Planned and Behavior (TPB), Protection Motivation Theory (PMT), the Health Belief Model (HBM), and the Stage of Change Model. Social cognition models emphasize assessing people’s behavior and their beliefs in a social context [19]. Although each model has a different emphasis, they have similar ideas on how people take action.

To explore factors that determine whether people practice social distancing behavior, this study uses the TPB [10]. The TPB is popular as it can be used to explain a wide-range of behaviors and can be applied to different populations and contexts [13, 21–26]. According to the TPB, behavior is predicted by intention, and intention is influenced by attitudes towards the behavior, subjective norms, and perceived behavior control (PBC). The more strongly an individual holds an attitude, subjective norm, or PBC towards a behavior, the more likely the person intends to perform the behavior. Based on this theory, attitude is the degree to which a person is in favor (or not) of a particular behavior. A person who accepts as true that performing a certain behavior will lead to a mostly positive outcome will hold a favorable attitude towards performing the behavior [10]. Social norms are a function of beliefs that most referents will approve and support a particular behavior, which will exert pressure on an individual to perform the behavior. Referents can be family members, religious leaders, friends, and teachers. Another component of the TPB is PBC. PBC refers to a person’s perception of whether there is an aid or obstacle to performing a given behavior [27].

Due to the widespread news during the COVID-19 outbreak, it is advantageous to include other variables related to the performance of health behaviors: risk perception and media use [28]. Previous studies that have applied the TPB have demonstrated an increasing predictive ability of the model by adding more TPB variables [29–32]. Risk perception, in particular, has been widely used to investigate protective behavior. A number of studies have concluded that disaster preparedness and health-risk behavior is influenced by risk perception [19, 33]. For example, the Health Behavior Model (HBM) theory posits that people will perform health behaviors in response to perceived susceptibility, severity, benefits, barriers, and cues to action [34]. Moreover, the integration of risk perception and the TPB has been found to be effective in studies of dental flossing [35], predicting safe food handling in adolescents [36], and the intention to take precautions to avoid consuming foods with additives [37]. However, few studies have been conducted that include risk perception in determining the components of the TPB.

The causal relationship between information and risk perception has also been studied. For instance, the effect of audience motivation and influence of news media on risk perception [38–40]. Most researchers agree that information and an individual’s level of knowledge can positively influence protective behavior [41]. For example, the results of a study on malaria prevention in Rwanda showed that providing timely malaria-related information improved the ability of people to control and eliminate the disease [42]. Likewise, the results of review on inaccurate communication regarding mortality caused by air pollution showed that shifting the focus away from blame to more accurate and clear information help people change their behaviors [43]. Another study that applied the extended TPB found evidence to support the important role of information in determining behavioral changes. However, the role of media use in influencing attitudes, social norms, and PBC are limited, especially in the context of a pandemic.

Other studies related to health behavior have investigated the role of demographic variables in determining health behavior. In particular, sex, age, and income are often used to explain variation in behavior. One study, conducted by Mniszewski and his colleagues [44], concerned the use of face masks during an epidemic in Southern California. They found that using masks was correlated with an individual’s age and sex, whereby females and older adults were more likely to wear a mask than males or youths. Other studies that have investigated the relationship between demographic variables and protective behavior support findings such as income differences concerning the prevention of SARS [45] and the association between age and preventative cardiovascular disease behaviors [46].

The purpose of this study was to explore people’s intention to practice social distancing during the COVID-19 pandemic based on the extended TPB. We proposed the following hypotheses:

1. Hypothesis 1 (H1): Attitude positively influences the intention to socially distance
2. Hypothesis 2 (H2): Subjective norms influence the intention to socially distance
3. Hypothesis 3 (H3): PBC positively influences the intention to socially distance
4. Hypothesis 4 (H4): Risk perception positively influences attitudes towards social distancing
5. Hypothesis 5 (H5): Risk perception positively influences subjective norms
6. Hypothesis 6 (H6): Risk perception positively influences PBC
7. Hypothesis 7 (H7): Media use positively influences risk perception
8. Hypothesis 8 (H8): Media use positively influences attitudes towards social distancing
9. Hypothesis 9 (H9): Media use positively influences subjective norms
10. Hypothesis 10 (H10): Media use positively influences PBC

We also explored demographic variables such sex, age and residential areas relative to the intension to socially distance. Previous studies have supported the idea that there are different behaviors between males and females, elder and young people, as well as whether one lives in a rural or urban area.

The TPB has been claimed to result in consistent findings across behavior categories [47] and different populations [10]. This study provides fundamental information on applicability of the TPB due to the specific social-cultural characteristic of the study population. Indonesians can be categorized as a collectivist culture in which the expression of the people is controlled by social norms [48]. According to [49], collectivist cultures focused more on feeling/emotion instead of logical thinking. It is also important to note that traditionally the informal leader plays an important role in the community. Indonesia has maintained a quasi-feudalistic character due to being ruled by kingdoms and sultanates before the colonial era [50]. It is interesting to comprehend whether such collectivism and patronage-client culture will result in a similar effect because one of the variables of the TPB is the subjective norm.

## Methods

### Aim and participants

As the aim of this study was to investigate people’s intention to practice social distancing during the COVID-19 pandemic based on the extended TPB, a cross-sectional study design was used. As social distancing policies were already enacted, a questionnaire (see Additional file 1) was designed and distributed to the sample population and collected through online-based media (google form) from March 26, 2020 to March 29, 2020. The questionnaire that consists of 23 questions and 14 variables was shared on social media (Facebook, Instagram and Twitter) and WhatsApp groups. Only people living in Indonesia who were at least 17 years of age were allowed to voluntarily participate. There were 1870 responses to the questionnaire. However, after data cleaning, only 823 questionnaires were analyzed. Cleaning includes checking the completeness of filled-in questionnaire, no double participant, and other criteria. Respondents answered questions about their age, sex, level of education, city of residence, information-seeking (media use) behavior, risk perception, attitudes toward social distancing, subjective norms, PBC, and their intentions to take social distancing actions. To protect private information of the participants, the questionnaire did not ask the name and address of the participants (anonymity).

### Measures

All of the extended TPB variables were measured using a 7-point scale (scored from 1 to 7) and most of the items were adopted from a previous study by Ajzen and Fishbein [10]. Risk perception was measured using 2 items: (i) “COVID-19 is a deadly disease”, and (ii) “I am likely infected with COVID-19”. However, the second item was dropped as Cronbach’s alpha and composite reliability did not meet the standard score. Attitudes regarding intention to perform social distancing were measured using 2 items: (i) “Spreading COVID-19 can be controlled by staying at home” and (ii) “Avoiding gatherings of large numbers of people and staying a minimum of 1 meter away from others will reduce the possibility of transmitting COVID-19”. Measures of subjective norms consisted of 3 items: (i) “my family (sons/daughter/wife/husband/parents/others) agree that I should stay at home to avoid being infected with COVID-19”, (ii) “my family (sons/daughter/wives/husband/parents/others) think I should stay at home to avoid being infected with COVID-19”, and (iii) “religious leaders support performing social distancing to avoid being infected with COVID-19”.

There were 3 items used for constructing perceived behavioral control: (i) “For myself, social distancing and avoiding large gatherings are easy to do”, (ii) “I can do anything while staying at home”, and (iii) “I have control over whether or not I see other people to prevent being infected with COVID-19”. Intention to perform social distancing consisted of 2 items: (i) “This week I am planning to stay at home and avoid meeting many people” and, (ii) “This week I will make an effort to stay at home and avoid meeting many people (and stay at least one meter away from others)”. Seven items were measured media information use: (i) television, (ii) radio, (iii) print, (iv) social media (Facebook, Twitter, Instagram, etc.), (v) domestic website, (vi) overseas website, and, (vii) WhatsApp groups. Demographic information included sex, age, and area of residence.

### Data analysis

Descriptive data were analyzed using the IBM SPSS Statistic 23.0 software package (IBM Corp., Armonk, NY, USA) and SmartPLS 3.3.2 (SmartPLS GmbH, Boenningstedt, Germany) was used for factor loading and structural equation analyses. The partial least squares (PLS) method is considered the most appropriate for prediction or exploratory modeling [51]. Other advantages of the method are the ability to include multiple dependent and independent variables, the ability to treat multicollinearity among the independent variables, and the ability to handle single-item measurements [52]. Therefore, PLS is a suitable tool to explore and predict people’s behavior regarding social distancing during the emergence of SARS-CoV-2 and for the application of the extended TPB model. The significant values of the structural model are set to *p*-value < 0.05.

## Results

### Respondent profiles and measurement models

The profiles of the respondents are shown in Table 1. There were 439 males (53.3%) and 384 females (46.7%). This sex’s proportion is similar to the national population with males about 50.2% and female 49.8% [53]. The majority of the respondents were between 26 and 55 years of age and had either undergraduate/diplomas (56.1%) or masters and/or doctoral degrees (33%). The highest percentage of respondents were government officers (51.9%). In regards to residence, 60.8% of the respondents resided on Java Island. Information on whether respondents had family members older than 65 years with heart and lung disease are also shown in Table 1.

**Table 1:** Demographic characteristics of the respondents

Characteristics	Frequency	Percent %
**Sex**	**823**	**100**
Male	439	53.3
Female	384	46.7
**Age**	**823**	**100**
17–25 years	89	10.8
26–35 years	206	25
36–45 years	247	30
46–55 years	221	26.9
56–65 years	59	7.2
> 65 years	1	0.1
**Educational level**	**823**	**100**
At least middle school	2	0.2
High school	87	10.6
Bachelor’s degree	462	56.1
Graduate degree	272	33
**Occupation**	**823**	**100**
Households	40	4.9
Student	62	7.5
Informal Sector	22	2.7
Private Employment	139	16.9
Government Officer	427	51.9
Others	133	16.2
**Living areas**	**822**	**100**
Urban	529	64.3
Rural	294	35.7
**Family members with high risk**	**–**	**–**
< 60 years old	491	60.6
Heart disease	558	69.5
Lung/respiratory disease	634	78.8

The responses of having family members with high risk applied more than one possibility answer. The percentage against the total respondents (*N* = 823)

To test the proposed hypotheses, it was essential to evaluate the reliability and validity of the latent variables before examining the structural model. Table 2 shows the results of factor loadings, Cronbach’s alpha, the composite reliability (CR), and the average variance extracted (AVE). Factor loading is preferred equal to or greater than 0.70, however if it is an exploratory research 0.4 or higher is acceptable [52]. Factor loading was set at a minimum of 0.6 [54] and the Cronbach’s alpha values were set at a minimum of 0.60 [54]. Internal consistency was evaluated and resulted in a composite reliability > 0.7. AVE reflects convergence and divergent validity and it is recommended that the threshold value for AVE should be exceed 0.5 [52]. The construct validity test is another discriminant validity, which aims to confirm that certain latent variables differ from others. The AVE square root was calculated and its value should be the highest in comparison with the correlations with other latent variables [55]. Table 3 shows the standard of discriminant validity. It can be seen from the Fornell-Larcker criterion table, the number of the square root of AVE appears in the diagonal cells are higher than the number correlations appear below it. Based on these results, the model was considered to be reliable, internally consistent, and with adequate discriminant validity.

**Table 2:** Mean, SD, Factor Loading, Cronbach’s Alpha, Composite Reliability and AVE

Construct and Items	Mean (1–7)	SD	Factor Loading	Cronbach’s α	CR	AVE
*Media Use*				0.856	0.887	0.755
Print	2.962	1.880	0.717			
Radio	2.544	1.703	0.627			
TV	4.400	2.120	0.814			
WhatsApp	5.146	1.907	0.775			
Social Media	4.925	2.020	0.749			
Domestic Web	5.094	1.882	0.771			
Overseas Web	3.547	2.068	0.629			
*Risk Perception*				1.000	1.000	1.000
Covid-19 is deadly	5.887	1.455	1.000			
*Attitude*				0.676	0.861	0.755
Stay-at-home	6.219	1.244	0.868			
Social distancing	6.495	0.685	0.870			
*Subjective Norm*				0.779	0.871	0.694
Family agrees	6.465	0.669	0.828			
Family support	6.417	1.016	0.880			
Religious leaders agree	6.340	1.029	0.789			
*Perceived Behavior Control*				0.751		0.663
Able to stay-at-home	5.360	1.656	0.769		855	
I can control	6.070	1.230	0.867			
Easy to stay at home	5.583	1.573	0.803			
*Intention*				0.864	0.937	0.881
Stay-at-home	5.614	1.609	0.939			
Socially distance	5.807	1.477	0.938			

*SD* Standard Deviation, *CR* Composite Reliability, *AVE* Average Variance Extracted, 1–7: Measurement scales

**Table 3:** Discriminant validity tests results (Fomell-Larcker Criterion)

Construct	ATT	Intention	Media	PBC	RP	SN
ATT	0.869*					
Intention	0.260	0.938*				
Media	0.107	0.009	0.729*			
PBC	0.418	0.367	0.041	0.814*		
RP	0.217	0.108	0.116	0.218	**Single Item**	
SN	0.628	0.382	0.104	0.511	0.285	0.833*

*ATT* Attitude, *PBC* Perceived behavior control, *RP* Risk perception, *SN* Social norm. Significance level: * *p* < 0.001, ** *p* < 0.05

### Structural model and hypotheses testing

**Fig. 1:**
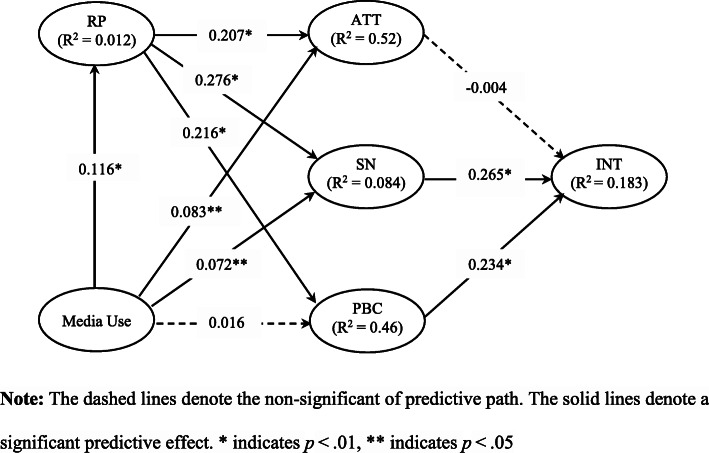
Structural Model Results. Note: The dashed lines denote the non-significant of predictive path. The solid lines denote a significant predictive effect. * indicates *p* < .01, ** indicates *p* < .05

To test the structural model and hypotheses, a bootstrapping procedure with 5000 iterations and 823 subsamples was used [56]. Figure 1 shows the results of the explained variance or adjusted R-square (R^2^) and path-values of the model. These results show that the R^2^ value of intention was 0.183, attitude was 0.052, social norms was 0.084, PBC was 0.046, and risk perception had value of 0.012. The R^2^ of intention was higher than the 0.10 threshold [57] indicating that 18.3% of the variance in social distancing intention can be explained by the components of the extended TPB.

Table 4 shows the assessment of the significance of the path coefficients, t-statistics and *p*-values among the components of the extended TPB model. Intention to socially distance was determined by social norms (β = 0.265, t-value = 4.575, *p*-value < 0.000) and PBC (β = 0.234, t-value = 4.625, *p* < 0.000). Risk perception significantly influenced attitudes (β = 0.207, t-value = 4.717, *p* < 0.000), social norms (β = 0.276, t-value **=** 6.333, *p* < 0.000), and PBC (β = 0.216, t-value = 5.418, *p* < 0.000). In addition, media use affected risk perception (β = 0.116, t-value = 3.177, *p* < 0.001), attitudes (β = 0.083, t-value = 2.323, *p* < 0.020), and social norms (β = 0.072, t-value = 2.158, *p* < 0.031). Therefore, hypotheses H2 through H9 were supported. Hypotheses H1 and H10 were not supported, as there were no significant causal relationships between attitudes and intentions, and media to PBC.

**Table 4:** Results of the proposed hypotheses test

Hypothesis	Path Coefficient (β-value)	t-statistic	***p***-value	Results
H1: ATT → INT	−0.004	0.068	0.945	Not Supported
H2: SN → INT	0.265	4.575	0.000*	Supported
H3: PBC → INT	0.234	4.625	0.000*	Supported
H4: RP → ATT	0.207	4.717	0.000*	Supported
H5: RP → SN	0.276	6.333	0.000*	Supported
H6: RP → PBC	0.216	5.418	0.000*	Supported
H7: Media → RP	0.116	3.177	0.001*	Supported
H8: Media → ATT	0.083	2.324	0.020**	Supported
H9: Media → SN	0.072	2.158	0.031**	Supported
H10: Media→ PBC	0.016	0.405	0.685	Not Supported

*ATT* Attitude, *INT* Intention, *PBC* Perceived behavior control, *RP* Risk perception, *SN* Social norm. Significance level: * *p* < 0.001, ** *p* < 0.05

### Multigroup analysis

As this study involved a wide range of respondent backgrounds, a multigroup analysis was used to explore whether media use, risk perception, and the TPB components were different across sexes (male vs. female), ages (elder vs. younger), and residential areas (urban vs. rural). A two-step procedure was used to examine the statistical differences between the groups under investigation: bootstrapping and Multi-Group Analysis (MGA). The bootstrapping procedure was used to assess the path coefficients and *p*-values of each group, as well as the mean, STDEV and t-values from the results. The PLS-MGA test assessed the differences in path coefficients and significant *p*-values between each group. A PLS-MGA test indicates significance differences between groups if the p-value is lower than 0.05 or larger than 0.95 for the differences between group-specific path coefficients [52].

Table 5 shows the result of the path coefficient for each group. H1 hypothesized that attitude positively influences the intention to socially distance, which was not supported across all of the groups. However, H2, which proposed that subjective norms influence the intention to socially distance, was supported across all of the groups: sex (male: β = 0.226, *p* < 0.05; female: β = 0.299, *p* < 0.001), age group (elder: β = 0.281, *p* < 0.05; younger: β = 0.263, *p* < 0.001), and residential area (urban: β = 0.262, *p* < 0.001; rural: β = 0.282, *p* < 0.05,). The third hypothesis, the influence of PBC on intention to socially distance (H3), was also confirmed for all of the groups: sex (male: β 0.310, *p* < 0.001; female: β = 0.153, *p* < 0.05), age group (elder: β = 0.357, *p* < 0.001; younger: β = 0.176 *p* < 0.001) and residential area (urban: β = 0.240, *p* < 0.001; rural: β = 0.229, *p* < 0.05). Similar to H2 and H3, the fourth hypothesis (H4), which suggested that risk perception influences attitudes towards social distancing, and H5, which suggested that risk perception influences subjective norms, was supported across all of the groups: sex (male: β = 0.263, *p* < 0.001 and β = 0.320, *p* < 0.001; female: β = 0.154, *p* < 0.05 and β = 0.227, *p* < 0.05), age group (elder: β = 0.191, *p* < 0.05 and β = 0.230, *p* < 0.05; younger: β = 0.221, *p* < 0.001 and β = 0.308, *p* < 0.001), and living area (urban: β = 0.164, *p* < 0.05 and β = 0.215, *p* < 0.001; rural: β = 0.299, *p* < 0.001). H7, which proposed that media use influenced risk perception, was supported in all of the groups: sex only for females (β = 0.127, *p* < 0.05), ages for the younger group (β = 0.127, *p* < 0.05) and residential areas for rural populations (β = 0.234, *p* < 0.05). The effect of media use on attitude, as hypothesized in H8, was only confirmed for the younger age group (β = 0.111, *p* < 0.05). H9 which suggested media use influences subjective norms, was supported only for females (β = 0.123, *P* < 0.05), while H10, which proposed media use positively influences PBC, was not supported in any of the groups.

**Table 5:** Multigroup analysis statistical tests

Construct	H1	H2	H3	H4	H5	H6	H7	H8	H9	H10
***Sex***										
**Male (*N*** **= 439)**	**0.021**	0.226**	0.310*	0.263*	0.320*	0.263*	**0.103**	**0.100**	**0.052**	**0.046**
**Female (*N*** **= 384)**	**−0.034**	0.299*	0.153**	0.154**	0.227**	0.161**	0.127**	**0.082**	0.123**	**−0.011**
**Diff.**	**0.055**	**−0.073**	**0.157**	**0.110**	**0.093**	**0.102**	**−0.024**	**0.018**	**−0.071**	**0.567**
**vPLS-MGA*p*-value**	**0.635**	**0.505**	**0.085**	**0.215**	**0.296**	**0.195**	**0.737**	**0.806**	**0.317**	**0.527**
***Ages***										
**Elder/>** **46 (*N*** **= 281)**	**−0.013**	0.281**	0.357*	0.191**	0.230**	**0.104**	**0.117**	**0.036**	**0.088**	**0.655**
**Younger/<** **45 (*N*** **= 542)**	**−0.002**	0.263*	0.176*	0.221*	0.308*	0.283*	0.127**	0.111**	**0.063**	**−0.121**
**Diff.**	**−0.010**	**0.018**	**0.181**	**−0.029**	**−0.077**	**− 0.178**	**−0.009**	**− 0.074**	**0.025**	**0.077**
**PLS-MGA p-value**	**0.949**	**0.872**	**0.056**	**0.724**	**0.375**	**0.020**	**0.995**	**0.459**	**0.668**	**0.390**
***Residential areas***										
**Urban (*N*** **= 529)**	**−0.033**	0.262*	0.240*	0.164**	0.215*	0.177**	**0.059**	**0.084**	**0.082**	**0.060**
**Rural (*N*** **= 294)**	**0.043**	0.282**	0.229**	0.299*	0.404*	0.305*	0.234**	**0.066**	**0.043**	**−0.087**
**Diff.**	**−0.076**	**−0.019**	**0.010**	**−0.136**	**− 0.188**	**−0.127**	**− 0.174**	**0.017**	**0.038**	**0.147**
**PLS-MGA*p*-value**	**0.623**	**0.858**	**0.909**	**0.135**	**0.028**	**0.108**	**0.014**	**0.819**	**0.562**	**0.082**

**H1…H10:** Hypotheses refer (same as) to the structural model of the extended TPB

* and **: Path coefficient each sub-group with significance level, * *p* < 0.001, ** *p* < 0.05

**Diff**: Path Coefficient Differences between sub-group in the PLS-MGA.

**Bold font:** PLS-MGA p-value below 5% and above 95% indicate a significant difference.

The PLS-MGA *p*-values indicated that there were no significant differences between sex. Multigroup analysis also indicated that there were small differences between the elder and younger age groups. Among the 10 hypotheses, only H6 and H7 were supported. H6 suggests that the effect of risk perception on PBC for young people (equal to and under 45 years old) was stronger than for elder people (difference = − 0.178, *p* < 0.05). H7 indicated that media use had more of an impact on risk perception for young people compare to elder people (difference = − 0.009, *p* > 0.095).

The differences between rural and urban residents applied to hypotheses H5 and H7. Hypothesis 5 (H5) described the effect of risk perception on social norms and was supported in rural populations (difference = − 0.188, *p* < 0.05). Similarly, H7 explained that the media’s effect on risk perception was also more effective for people living in rural areas (difference = − 0.174, *p* > 0.05).

## Discussion

Out of the 3 components of the TPB, social norms and PBC were significant in determining the intention to socially distance, while attitude was not a significant predictor of intention. These findings are in agreement with a previous study that found subjective norms and PBC had a significant impact on behavioral intentions [58–60]. In line with the findings of Alfahan’s study [61], the social norm component emerged as the most important predictor. Family members and religious leaders were important in influencing people’s intention to practice social distancing to reduce the spread of SARS-CoV-2. One possible reason for this finding could be due to the behavioral style of Indonesians, which is grouped into 3 categories: (1) sociable community-oriented (70%), (2) positive but still self-oriented (27%), and (3) self-centric (3%) [62]. This implies that effective policy interventions to influence behavior to include more social distancing would be to involve religious and community leaders, and large families. As found in a study conducted by Rajib Shaw and his colleagues [63] people in China, Japan, and South Korea also made decisions that strongly depended on community solidarity and behavior.

The contribution of the PBC variable to the model indicates that people have control and the ability to take social distancing intention. This finding supports past TPB studies that have found PBC variables are typically less important than attitudes and social norms [64] [47, 65, 66];. Contrary to our expectations, attitude was not predictor of intention. This finding is in contrast to previous studies on alcohol consumption and smoking cessation, which have concluded that attitude was a predictor of intention [20, 67]. One possible reason for this result is that individuals may believe that reducing the spreading of the virus is not limited to staying at home and avoiding crowds, as questioned in the questionnaires. For example, to reduce the spread SARS-CoV-2, WHO suggests that regularly washing hands using soap and water or cleaning with alcohol-based hand sanitizers, covering the mouth and nose when coughing, and consuming healthy foods be included with social distancing. As argued by Ajzen and Fishbein [10] the relative importance of the TPB components for the prediction of behavior intention diverges depending on the target behavior and population.

The role of risk perception in protective behavior has been widely studied, and its effect on social norms, risk perception and PBC components are important. Risk perception can predict behavioral intentions mediated by attitudes, social norms, and PBC. The present results are consistent with a previous study in which risk perception was a strong determining factor for attitudes and self-efficacy in a health context [35]. These findings suggest that it is critical to identify the level of risk perception of COVID-19 when social distancing is promoted and intervention policies are formulated. Policymakers should consider applying behavioral promotions that increase risk perception. To our knowledge, this the first study to include risk perception as influencing the components of TPB in the context of global health crisis in less-developed country.

The influence of media use on risk perception and the intention to enact behavior was also investigated. Media use significantly changed risk perception, attitudes, and social norms but not the PBC variable. Similar to risk perception, most studies have focused on the direct effects of communication variables on behavioral change, and these have resulted in mixed findings [13, 68]. This study used communication variables as predictors of risk perception, attitudes, and social norms, and the results were significant. This finding supports the elaboration of the likelihood model and suggests that attention to message content is a necessary condition for persuasive effects [69]. These results also suggest that both print and digital media influence the level of risk perception, attitudes, and social norms when predicting social distancing practices to prevent the spreading of the COVID-19.

In addition, the present study found minor differences in demographic characteristics such as sex, age, and area of residence. In contrast to previous studies [64, 70], we found no differences between males and females in how behavioral change is influenced. These findings are inconsistent with past studies that found differences in behavioral intention between the sex [71]. It may be due to the wide media coverage reporting on COVID-19 causing both males and females to receive and respond to social distancing recommendations equally. Factors related to media and its social influence may be significant in the context of a pandemic [72]. Therefore, it is necessary to consider the media’s role in planning effective risk communication.

Age and residential area showed partial differences. Younger respondents demonstrated a stronger relationship between the influence of risk perception and PBC as well as media use and risk perception. This finding is consistent with a previous study that found PBC associated with self-efficacy and that control ability was more easily acquired by younger individuals [30]. The relationship between risk perception and perceived behavioral control, and media use and risk perception for people living in rural areas was greater than those living in urban areas. This finding contrasts with a previous study that found people living in the city were more likely to buy green products due to a greater availability of information than those living in rural areas [73]. In the case of COVID-19, people in rural areas may often only use official, government media compared to urban residents, who also use social media or WhatsApp groups—where the information may not be valid. This finding also supports the negative association between media use and perceived behavioral control, which was significant for people living in rural areas.

## Conclusions

This study included an examination of risk perception and media use in the extended TPB model to predict the public’s intention of practicing social distancing during the SARS-CoV-2 pandemic. The empirical results of a partial least squares structural equation modeling analysis with 823 participants revealed that the extended TPB model supported 8 out of 10 proposed hypotheses. Subjective norms and PBC significantly influenced the intention to practice social distancing, while attitude was not significantly associated with intention. Risk perception significantly affected attitudes, subjective norms, and PBC. Furthermore, while media use influenced risk perception, attitudes, and subjective norms, it was not a significant factor in influencing PBC. A multigroup analysis of demographic variables found partial differences. Younger individuals showed a stronger relationship between the influence of risk perception and PBC and media use on risk perception. In addition, the predictive strength of subjective norms from risk perception and risk perception from media use was more strongly associated with rural populations.

The results of current study provide a foundational understanding of the level of the public’s risk perception such that it can be used to target policy interventions needed to reduce the spread of SARS-CoV-2. Our findings also revealed the role of media use in determining risk perception, attitudes and subjective norms. Better and effective risk communication can change the level of risk perception, attitudes and subjective norms and, in turn, change people’s intention to socially distance. This study can be a valuable addition to the literature, as it explores the notions of behavioral changes in the context of a large infectious disease pandemic especially in developing countries context. While this study supported the extended TPB, the collection of data through social media was a limitation. Therefore, future studies should be expanded to include random population sampling. Another limitation of the study is that the non-significant differences might be caused by demographic factors such as sex, age, educational background, and marital status. Furthermore, since the characters of the population are collectivist cultures, the roles of risk perception, media use, emotional and environmental factors contribute to the non-significant differences. Future studies should investigate in-depth the contribution of demographic and social factors of the participants. It will also critical to investigate the effect of the government’s risk communication on public behavior in the context of a pandemic.

## Supplementary Information

**Additional file 1.**

## Data Availability

The data used in this this study are available from the corresponding author upon reasonable request.

## Abbreviations

AT: Attitude
AVE: Average Variance Extracted
COVID-19: Coronavirus disease 2019
CA: Cronbach’s alpha
CR: Composite Reliability
INT: Intention
MGA: Multigroup Analysis
PBC: Perceived Behavior Control
TPB: Theory of Planned Behavior
PLS: Partial Least Squares
RP: Risk Perception
SARS-CoV-2: Severe acute respiratory syndrome coronavirus 2
SD: Standard Deviation
SN: Subjective Norms
SPSS: Statistical Package for the Social Sciences
WHO: World Health Organization
