# Supplementary material for: Social distancing intentions to reduce the spread of COVID-19: The extended theory of planned behavior
Source: BMC Public Health. 2021 Oct 11;21:1836. doi: 10.1186/s12889-021-11884-5 (PMC8503732; doi:10.1186/s12889-021-11884-5)
Supplement: Supplementary file 1 — Additional file 1. [file 12889_2021_11884_MOESM1_ESM.pdf]

Additional File 1

Survey of Perception and Information on Covid-19 (Corona)

Dear ladies and gentleman

We are from a team of Research Centre for Conflict and Policy (RCCP Faculty of Administrative Science, Brawijaya University) wish to have support from all of you to fulfill questionnaires regarding perception, attitude response and behavioral use of information sources concerning Covid-19 pandemic spreading in Indonesia.

The results of the survey will be used to contribute to policy recommendations for the government or other stakeholders. The results of the study (research or policy paper) will also be possible to be published. All information of respondents provided in this questionnaire will be kept confidential.

If you are an Indonesian citizen and living in the Indonesia territory, above 17 years-old, and with awareness and voluntarily want to participate in this survey, please complete the questionnaires below.

Thank you for your contribution. We expect it will contribute to better policies in responding to the pandemic in the future.

Best Regards

Wilopo
Wignyo Adiyoso

* Required

PLEASE CHOOSE EVERY SINGLE STATEMEN BELOW

1. COVID-19 is a deadly disease *

Mark only one oval

	1	2	3	4	5	6	7	
Strongly Disagree	⬭	⬭	⬭	⬭	⬭	⬭	⬭	Strongly Agree

2. I am likely infected with COVID-19 *

Mark only one oval.

	1	2	3	4	5	6	7	
Strongly Disagree	⬭	⬭	⬭	⬭	⬭	⬭	⬭	Strongly Agree

3. Spreading COVID-19 can be controlled by staying at home *

Mark only one oval.

	1	2	3	4	5	6	7	
Strongly Disagree	◯	◯	◯	◯	◯	◯	◯	Strongly Agree

4. Avoiding gatherings of large numbers of people and staying a minimum of 1 meter away from oth will reduce the possibility of transmitting COVID-19 *

Mark only one oval.

	1	2	3	4	5	6	7	
Strongly Disagree	◯	◯	◯	◯	◯	◯	◯	Strongly Agree

5. My family (sons/daughter/wife/husband/parents/others) agree that I should stay at home to avoid being infected with COVID-19 *

Mark only one oval.

	1	2	3	4	5	6	7	
Strongly Disagree	◯	◯	◯	◯	◯	◯	◯	Strongly Agree

6. My family (sons/daughter/wives/husband/parents/others) think I should stay at home to avoid bein infected with COVID-19 *

Mark only one oval.

	1	2	3	4	5	6	7	
Strongly Disagree	◯	◯	◯	◯	◯	◯	◯	Strongly Agree

7. Religious leaders support performing social distancing to avoid being infected with COVID-19 *

Mark only one oval.

	1	2	3	4	5	6	7	
Strongly Disagree								Strongly Agree

8. For myself, social distancing and avoiding large gatherings are easy to do *

Mark only one oval.

	1	2	3	4	5	6	7	
Strongly Disagree								Strongly Agree

9. I can do anything while staying at home *

Mark only one oval.

	1	2	3	4	5	6	7	
Strongly Disagree								Strongly Agree

10. I have control over whether or not I see other people to prevent being infected with COVID-19 *

Mark only one oval.

	1	2	3	4	5	6	7	
Strongly Disagree								Strongly Agree

11. Since the pandemic emergency declared by the government (17/03/2020), did you stay (isolated at home .. *

Mark only one oval.

- Yes, I stayed at home at all the time
- Yes, BUT I frequently did activities outsideNO,
- I did daily activities as before

12. If you responded "Yes, BUT I frequently did activities outside", what kind activities you did mostly (per day)

Check all that apply.

	Not at all	1 times	2 times	More than 3 times
Working	☐	☐	☐	☐
Shoping daily needs	☐	☐	☐	☐
Meeting other families	☐	☐	☐	☐
Recreation/leisures times	☐	☐	☐	☐
Others	☐	☐	☐	☐

13. This week, I am planning to stay at home and avoid meeting many people *

Mark only one oval.

	1	2	3	4	5	6	7	
Strongly Disagree	◯	◯	◯	◯	◯	◯	◯	Strongly Agree

14. This week, I will make an effort to stay at home and avoid meeting many people (and stay at leas one meter away from others *

Mark only one oval.

	1	2	3	4	5	6	7	
Strongly Disagree	◯	◯	◯	◯	◯	◯	◯	Strongly Agree

MEDIA AND SOURCE OF INFORMATION

15. Information media and frequently on Covid-19 pandemic which I read, listen, watch and discuss

Check all that apply.

	Never	Almost never	Rarely	Sometimes	Often	Almost	Alway
Television							
Newspaper (printed media)							
Website/Internet							
Radio							
FB, IG, Twitter, youtube,google dll							
Whatsup Group							
Media for overseas							
Langsung (antar orang)							

16. Sources of Information (authorities provided information) of Covid-19 in which I trusted are ...

Check all that apply.

	No trust at all	No trust	Less trust	Almost trust	Tust	More trust	Strongl trust
Central Government							
Local Government							
Experts/Academisi/Researchers							
Religious Leaders							
International Organization							
People who experincing (affecting) Covid-19							

17. Issues on Covid-19 pandemic that become your concerns are ...

Check all that apply.

	Never	Almost never	Rarely	Sometimes	Often	Almost	Alway
Government policies on how torespond pandemic	☐	☐	☐	☐	☐	☐	☐
The progress of the number people affected, recovery and die	☐	☐	☐	☐	☐	☐	☐
How to protect and to respond Covid-19	☐	☐	☐	☐	☐	☐	☐
Economy impact of Covid-19	☐	☐	☐	☐	☐	☐	☐
Social and religions issues	☐	☐	☐	☐	☐	☐	☐
Any news from overseas	☐	☐	☐	☐	☐	☐	☐

ABOUT ME

18. My age...*

Mark only one oval.

- 17 - 25 years
- 26 - 35 years
- 36 - 45 years
- 46 - 55 years
- 56 - 65 years
- Above 65 years

19. Gender *

Mark only one oval.

- Male
- Female

20. Do you have any family members living with you (excluding you) who ...

Mark only one oval per row.

	Yes	No	Do not know
Elderly people or > 65 years			
Having heart desease/hipertensionsHaving			
lung or respiratory disease			

21. Education level *

Mark only one oval.

- Under junior school
- Senior High School
- Diploma or Undergraduate
- Post-graduate (master or doctor)

22. Main job *

Mark only one oval.

- Students
- Housewife
- Informal sectors
- Private company
- Government officers
- Others

23. I am currently living in *

Mark only one oval.

- City
- Rural

24. I am living in Province .. *

Mark only one oval.

- DKI Jakarta
- Banten
- Jawa Barat
- Jawa Tengah
- DI Yogyakarta
- Jawa Timur
- Bali
- NTT atau NTB
- Di Pulau Sumatera
- Di Pulau Kalimantan
- Di Pulau Sulawesi
- Di Pulau/Kepulauan MalukuDi
- Pulau Papua
- Other: ______________________

This content is neither created nor endorsed by Google.

Google Forms
